# A Randomized Crossover Intervention Study on the Effect a Standardized Maté Extract (*Ilex paraguariensis* A. St.-Hil.) in Men Predisposed to Cardiovascular Risk

**DOI:** 10.3390/nu13010014

**Published:** 2020-12-23

**Authors:** Karimi S. Gebara, Arquimedes Gasparotto Junior, Rhanany A. C. Palozi, Christine Morand, Carla I. Bonetti, Paula T. Gozzi, Martha R. F. de Mello, Telma A. Costa, Euclides L. Cardozo Junior

**Affiliations:** 1 Laboratory of Electrophysiology and Cardiovascular Pharmacology, Health Sciences College, Federal University of Grande Dourados, R. João Rosa Góes, 1761, Dourados CEP 79825-070, MS, Brazil; karimi_sater@yahoo.com.br (K.S.G.); arquimedesgasparotto@gmail.com (A.G.J.); rhananypalozi@hotmail.com (R.A.C.P.); 2 Human Nutrition Unit, Université Clermont Auvergne, INRAE, F-63003 Clermont-Ferrand, France; christine.morand@inrae.fr; 3 Institute of Biological, Medical and Health Sciences, Universidade Paranaense, Av. Parigot de Souza, 3636 J. Prada, Toledo 85903-170, PR, Brazil; carlaabonetti@gmail.com (C.I.B.); paula_gozzi@hotmail.com (P.T.G.); gu.gu_mello@hotmail.com (M.R.F.d.M.); telmacosta1971@gmail.com (T.A.C.)

**Keywords:** clinical trial, cardiometabolic disease, maté extract, chlorogenic acids, caffeoyl quinic acids, fasting glucose, *Ilex paraguariensis*

## Abstract

(1) Background: Due to its richness in chlorogenic acids (CGAs), Maté (*Ilex paraguariensis* A. St.-Hil.) could be of interest in the prevention of cardiometabolic diseases, however clinical evidence are lacking. This trial aimed to evaluate the impact of maté CGAs, consumed in a daily dose achievable through traditional maté beverages, on parameters related to cardiometabolic risk. (2) Design: Thirty-four male volunteers aged 45–65 years and with at most one criteria of metabolic syndrome, were recruited for a randomized, double-blind, placebo-controlled, and crossover study. The volunteers were assigned to consume an encapsulated dry maté extract for four-weeks, providing 580 mg of caffeoyl quinic acid derivatives (CQAs) daily, or a placebo, with a two weeks washout between intervention periods. Anthropometric variables, blood pressure, plasma glucose, lipids, endothelial, and inflammatory biomarkers were measured in overnight-fasted subjects and after a glucose load. (3) Results: We found no significant effects of treatment on these parameters and the response to the glucose load was also similar between the two interventions. However, a significant decrease in fasting glucose was observed between day 0 and day 28 for the maté group only (−0.57 ± 0.11 mmol/L, *p* < 0.0002). In subjects with an intermediate to high Framingham risk score, consumption of maté extract induced a 10% increase of high-density lipoprotein (HDL)-c from baseline. In a subgroup representative of the study population, significant decreases in the C-reactive protein (CRP) (−50%) and interleukin-6 (IL-6) (−19%) levels were observed. (4) Conclusions: These clinical observations suggest that maté, naturally rich in CGAs, could improve some cardiometabolic markers in subjects with a higher predisposition to metabolic syndrome, even if that remains to be confirmed in new trials specifically targeting this population.

## 1. Introduction

Plant based diets are known as the most protective for our health, especially by reducing the incidence of cardiometabolic diseases [1]. A growing body of epidemiological evidence explains the contribution of polyphenols, a category of bioactive compounds that are specifically found and abundant in plant foods, to these protective effects [2]. Current evidence from clinical and pre-clinical studies indicates that polyphenol intake could relieve the metabolic syndrome components by decreasing body weight, blood pressure, blood glucose and improving lipid metabolism [3,4].

Dietary polyphenols include a variety of flavonoids and non-flavonoid compounds, the latter being mainly phenolic acids. Due to their high content in foods widely consumed around the world, such as coffee, tea, cereals, and fruits, phenolic acids are the main contributors to the daily intake of polyphenol [5]. The health effects of phenolic acids have been less investigated than those of flavonoids. However, chlorogenic acids (CGAs), which are particularly abundant in coffee, have recently gained increasing attention with respect to their potential cardiovascular-preserving effects [6]. CGAs comprise a large family of esters formed between caffeic and quinic acids, leading to different subgroups that include caffeoylquinic, p-coumaroylquinic and feruloyquinic acids [7]. The absorption of CGAs is relatively low. Approximately one third of the ingested CGAs are absorbed in the upper gastrointestinal tract, the rest reaching the colon to be intensely metabolized by microbiota which releases a range of microbial catabolites which can then be absorbed [7]. 

The physiological and biochemical effects of CGAs on a number of biomarkers related to cardiometabolic diseases have been recently reviewed [8]. In acute studies, CGAs have been shown to improve glucose tolerance in healthy subjects [9,10] and increase insulin sensitivity in patients [11]. Chlorogenic acid in coffee bean extract has been reported to decrease blood pressure in hypertensive humans [12,13] and to improve acutely endothelial function in healthy subjects under baseline conditions [14] or after glucose load [15]. 

Maté (*Ilex paraguariensis* A. St.-Hil.), which is being increasingly considered for its potential as a functional food [16], is another food especially rich in CGAs. The peculiarity of maté is that it contains mainly mono-caffeoyl quinic acids (mono-CQAs), including 3-caffeoylquinic, 4-caffeoylquinic and 5-caffeoylquinic acids, and di-caffeoyl derivatives (di-CQAs), including 3,5-dicaffeoylquinic, 4,5-dicaffeoylquinic, and 3,4-dicaffeoylquinic acids [17]. In different animal models of metabolic syndrome, supplementation studies with aqueous maté extract showed improvement in the blood lipid profile [18,19,20], and glucose and insulin homeostasis [20]. After maté supplementation, an attenuation of the development of atherosclerosis was also observed in cholesterol fed rabbits [21] and a reduction of the inflammation reported in obese rats [22]. In humans, the consumption of maté tea or extracts was reported to reduce the percentage of body fat in overweight subjects [23] and the biomarkers of oxidative stress in normo and dyslipidemic subjects [24]. Consumption of maté tea infusion has been also shown to improve glycemic control in diabetic and prediabetic subjects [25] and to ameliorate the lipid profile of dyslipidemic subjects [26]. These benefits could be attributed mainly to maté CGAs; however, clinical evidence is still very limited. 

This present study aimed to evaluate the sub-chronic effect of a consumption of an CGAs rich maté extract on a range of anthropometric, hemodynamic, metabolic, and inflammatory parameters related to cardiometabolic risk, through a double blind, randomized, crossover study performed on healthy overweight, middle-aged men with a free life. We selected a representative population with predisposition to cardiometabolic risk, but without diagnosed or treated pathology, reflecting the increased prevalence of adverse metabolic risk factors in the Brazilian adult population [27]. At the same time, it reflects a representative group of consumers of the maté traditional beverage [28]. Further, we excluded women to avoid any interference of the hormonal status (menstrual cycle) on the response to dietary intervention. Volunteers had to consume a standardized maté extract for four-weeks, providing phenolic compounds in easily achievable amounts through the consumption of maté traditional beverages.

## 2. Materials and Methods

### 2.1. Subjects

Thirty-five male volunteers, aged 45–65 years, were recruited for the study. All subjects had no more than one of the five criteria associated with metabolic syndrome proposed by the National Cholesterol Education Program’s Adult Treatment Panel III (NCEP-ATP III) and approved by Brazilian scientific societies in the First Brazilian Guideline for Diagnosis and Treatment of Metabolic Syndrome (2005). For men, these criteria include waist circumference above 40 inches, blood pressure above 130/85 mmHg, fasting triglyceride (TG) level above 1.70 mmol/L, fasting high-density lipoprotein (HDL) cholesterol level less than 1.04 mmol/L, and fasting blood sugar above 5.55 mmol/L.

**Figure 1:**
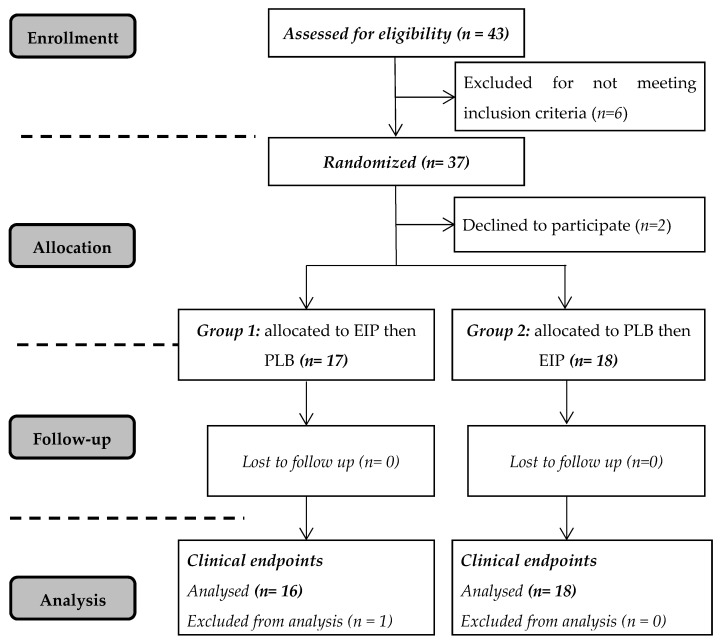
Diagram of flow of participants during the study. EIP = *Ilex paraguariensis* extract; PLB = placebo.

Exclusion criteria included antioxidants or vitamin supplements in the last 3-months prior to the study; smokers or individuals that have quit smoking for less than three years; chronic alcoholism; diagnosed with chronic diseases (diabetes, hypercholesterolemia, severe hypertension, mental illness and renal, or hepatic disease) and receiving associated medications. The study was approved by the Human Ethics Committee (CEPEH) of the Paranaense University (CAAE: 22531313.5.0000.0109). All volunteers signed a Consent Form, thirty-five completed the study, but one was excluded from the analysis for not following the guidelines during the protocol. As a result, thirty-four volunteers were evaluated; see Figure 1. This study was registered at www.clinicaltrials.gov under Protocol ID UNP-ILCV-1518.

### 2.2. Preparation and Analysis of Maté Extract

The goal was to produce a standardized maté extract to be administered daily, with a dosage of CQAs similar to that provided by the consumption of traditional maté beverages. That has been previously reported to range from 510 to 1700 mg/d [28]. The extract was supplied by the company SUSTENTEC (www.sustentec.org.br). The extract was prepared by infusion of dry maté leaves and drying in spray-dryer (ratio 5:1 leaves/extract). 

**Figure 2:**
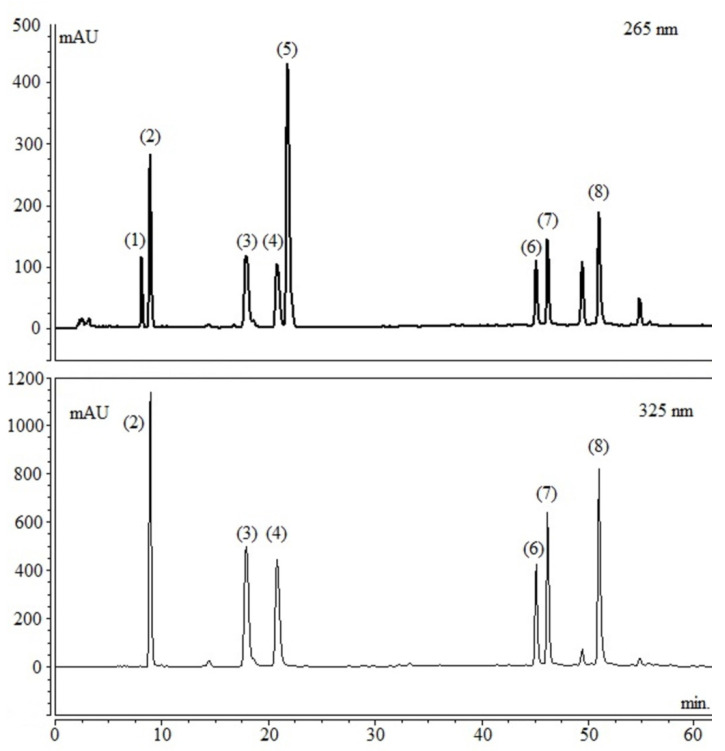
Representative high performance liquid chromatography coupled to a diode detector (HPLC-DAD) chromatogram of *Ilex paraguariensis* extract (EIP), signal at 265 and 325 nm. The compounds indicated are (1) theobromine, (2) 3-*O*-caffeoylquinic acid (3-CQA), (3) 5-*O*-caffeoylquinic acid (5-CQA), (4) 4-*O*-caffeoylquinic acid (4-CQA), (5) caffeine, (6) 3,4-di-*O*-caffeoylquinic acid (3,4-diCQA), (7) 3,5-di-*O*-caffeoylquinic acid (3,5-diCQA), and (8) 4,5-di-*O*-caffeoylquinic acid (4,5-diCQA).

The phytochemicals content, including CGAs derivatives, caffeine, and theobromine, in the resulting dry extract was analyzed (Figure 2) and quantified (Table 1) by high performance liquid chromatography coupled to a diode detector (HPLC-DAD—Varian—PRO STAR Mod. 210). The column used was the reverse phase C-18 (Phenomenex LUNA, 250.0 × 4.6 mm × 5.0 μm) for chromatographic separation. The elution system consisted of acidified water with 0.3% acetic acid, purified by the Milli-Q system (Merck Millipore, Milford MA, USA) (A) and methanol (B) (J.T. Baker), gradient 15–20% B for 20 min, 20–40% B for 25 min, 40–85% B for 50 min, and 85–15% B for 10 min, flow rate 1.0 mL/min. The column was maintained at 25 °C. Detection was monitored at 265 nm for methylxanthines and 325 nm for chlorogenic acids.

Based on data obtained from the quantification of CQAs in the extract and considering the minimal daily dose of total CQAs ingested (about 510 mg/day) through the consumption of traditional maté as reported by Gebara et al., 2017 [28], the daily administered dose of maté extract in the study was set at 2250 mg which provided 581 mg CQAs. Di-caffeoylquinics accounted for about 54% of the CQAs present in maté extracts and 46% were mono-caffeoylquinic acids (Table 1). This standardized maté extract (EIP) was used for encapsulation (250 mg dry extract/capsule) and to reach 581 mg CQAs/d, nine capsules have to be consumed daily. Each placebo (PLB) capsule contained only 250 mg of starch. 

### 2.3. Study Intervention

The main objective of the study was to evaluate the sub-chronic effect of maté extract consumption on some anthropometrics and clinical markers associated with cardiometabolic risk. For this, a 2-arms controlled, randomized, crossover trial was conducted over 4-weeks for comparing effects with maté extract (EIP) versus placebo (PLB). Two weeks of wash-out were applied between treatments. The order of administration of the product was randomized by an independent person from outside the clinical study, who was also responsible for handling the capsules. The person in charge of blood sampling, measurement and analysis was blinded to the allocation of treatments. The total duration of the study per volunteer was 12 weeks, including the inclusion phase (2 weeks), two experimental periods of 4-weeks each and a wash-out period (2 weeks). 

The volunteers were evaluated for inclusion, where parameters such as blood cells count, urea, hepatic enzymes, lipid parameters, fasting blood glucose, and anthropometric and hemodynamic parameters (waist circumference, BMI, pulse, and blood pressure) were evaluated. After their inclusion, the volunteers made 4 visits to the clinical research unit, on the first (V1 and V3) and last (V2 and V4) days of each experimental period. Anthropometric, blood pressure measurements, and blood sample collection were performed at each visit. During the entire study period, participants were asked to maintain their lifestyle and their dietary habits except that they were instructed to completely refrain from consuming maté products (chimarrão, terere) and to limit their total intake of polyphenol rich beverages (tea, coffee, cocoa, wine, soy milk, and fruit juices) to 200 mL/day. Before the beginning of each treatment period, the capsules of maté or placebo were distributed to volunteers who were instructed to consume three capsules every eight hours, from morning to evening (3 × 3 caps/day, providing a total of 2,250 mg of dry extract or placebo), for 28 days. The consumption of the capsules was monitored, a daily record was provided to volunteers at the beginning of each period to report their daily intake of study products. Volunteers had to bring back the record at the end of each period (V2, V4). Adherence to the intervention was assessed by examining the daily records and counting the capsules not consumed.

At each study visit (V1 to V4), the overnight-fasted subjects (*n =* 35) arrived at the clinical research unit approximately at 7:00 a.m., and after a 10 min rest period, systolic (SBP) and diastolic blood pressure (DBP), pulse (PUL) were measured and then waist circumference (WC) and body weight (BW). After that, a blood sample was collected to evaluate biochemical characteristics: the fasting glucose (GLU), total cholesterol (TC), HDL-cholesterol (HDL-c), LDL-cholesterol (LDL-c), and triglycerides (TGY). At the end of each experimental period (at V2 and V4), an oral glucose tolerance test (OGTT) was also performed. A standard dose of 75 g of dextrose diluted in water was ingested orally and glucose blood levels were checked at the time (T) = 0, 0.5, 1.0, 1.5, 2.0, and 3.0 h after the intake. Then volunteers then received a light meal before leaving the clinical unit. 

For a subgroup of subjects (*n* = 12), in order to maintain representative phenotypic characteristics in relation to cardiovascular risk, some inflammatory mediators were also evaluated in fasting (V1, V2, V3, and V4) and after the glucose tolerance test (V2, V4), those included: C-reactive protein (CRP) at T + 1.0 h, intercellular adhesion molecule 1 (ICAM-1), vascular cell adhesion molecule 1 (VCAM-1) at T + 2.0 h, and interleukin-6 (IL-6) at T + 3.0 h. 

### 2.4. Blood Pressure Measurements 

Measurements were taken in the morning after an overnight fasting in a quiet temperature-controlled room (22–25 °C). The subjects were placed silently for 10 min. In a sitting position before SBP and DBP were measured on the right upper arm with a validated and automated sphygmomanometer device (G-Tech, MA100) and PUL was monitored manually at the same time. Three consecutive measurements were recorded at 5-min intervals. The average of these consecutive SBP and DBP readings and the last PUL measured were considered for statistical analysis. BW was measured on a calibrated digital scale, used throughout the study period, and the WC was measured with a metric tape. During the study period, the same trained operator who was blind for treatment allocation performed each of these measurements.

### 2.5. Sampling and Analysis of Biological Samples

For the collection of hemogram, total and fractions cholesterol, glucose/insulin, tubes with anticoagulant-EDTA, separator gel and fluoride-EDTA were used, respectively. After blood sampling, the plasma were immediately isolated and stored at −80 °C until analysis. Plasma glucose concentrations, triglycerides, total and fractions cholesterol, and the hemogram were measured according to standardized laboratory procedures. The results of the oral glucose tolerance test were analyzed by applying the trapezoidal rule for the area under the curve calculation (AUC) according to Regnault et al. [29]. 

The concentrations of plasma inflammatory cytokines and endothelial activation biomarkers were assayed by ELISA (iMark™, Microplate Absorbance Reader, Bio-Rad Laboratories Inc., São Paulo, Brazil) following the manufacturer instructions using kits from Thermo Scientific for sICAM-1 and Invitrogen for IL-6 and sVCAM-1. CRP was performed on Roche COBAS Integra 400 plus. 

### 2.6. Statistical Analyses

The sample size was calculated according to primary endpoints, to achieve a significant difference between groups (>5%). Based on measurements of glucose, total cholesterol, SBP and DBP performed with a SD of the means of 0.98 mmol/L, 0.98 mmol/L, 17.6 mmHg, and 14.2 mmHg respectively to observe a difference of 10%, the sample size calculation indicated that for the more stringent condition (cholesterol) a number of 33 subjects was requested to complete the study (alpha = 0.05, power of 90%). In order to overcome potential drop-out during study, 35 volunteers were recruited.

The variables are presented as means ± SD. Data from the outcome variables were tested for normality and log-normality using the Shapiro–Wilk test. The PROC MIXED (version 9.0; SAS Institute Inc, Cary, NC, USA) procedure was used to analyze the fixed effects of dietary treatment on all the measured variables. In this model, the effects of subjects were included as random effects and the interaction between treatment and period was treated as the fixed effects. The data at the end of treatment were analyzed by mixed linear regression model, with baseline measurement being covariate. A significant main effect of treatment is observed when the P value of the F test for the main effect was <0.05. When analysis of covariance showed a statistically different main effect, least-squares-means comparisons (adjusted for multiple comparisons by the Tukey–Kramer method) were carried out to identify differences between pairs of treatment means. The PROC MIXED procedure was used to analyze carryover effects from one diet period to the next. 

No carryover effect (order of treatment, interaction between period, and treatment) was observed for any of the measured outcomes. Data quality control (prior to covariance analysis) was managed using individual value plots and I-charts in software Minitab, version 17. A subject was deleted after data quality control performed by Minitab software. This was developed in order to check for any outliers in the data set in different periods of sampling. The paired Student’s t-test was applied to verify the effects of maté consumption on inflammatory mediators. Values of *p* < 0.05 were considered significant. 

## 3. Results

### 3.1. Baseline Characteristics of Subjects at Inclusion and Compliance

Table 2 summarizes the inclusion values for the study population, including means with standard deviations and ranges obtained for each evaluated parameter. Considering the baseline inclusion values, the study population was generally about 50 years old, overweight (mean BMI = 27.28), normotensive, normoglycemic, and slightly dyslipidemic, mainly based on cholesterol values. The lipidemia ranged from normal to hyperlipidemic for total cholesterol and triglycerides (16 being normal and 18 hyperlipidemic). In the studied population, the mean Framingham risk score (FRS), which reflects the risk to develop cardiovascular disease in the next 10 years was of 11%. According to their FRS values, nineteen volunteers were classified as low risk (<10%), twelve as intermediate risk (10 to 20%) and three as high risk (>20%). Population values for classical hematological, renal, and hepatic parameters were normal (data not showed) and participants were overall healthy and without medication. Based on the examination of the daily record filled by volunteers with the number of capsules consumed and the number of capsules they brought back, the compliance of volunteers has been estimated at 97%.

### 3.2. Anthropometric, Hemodynamic and Biochemical Parameters

All these parameters were evaluated in the state of fasting at the initial and final visit of each intervention period, with an interval of four-week (Table 3). When compared to the placebo group, the intervention with EIP did not result in statistically significant changes in the anthropometric and hemodynamic data (WC, BW, BMI, SBP, DBP, and PUL) nor in the blood lipid profile, including Total Cholesterol, LDL-cholesterol, HDL-cholesterol, and Triglycerides values. 

**Figure 3:**
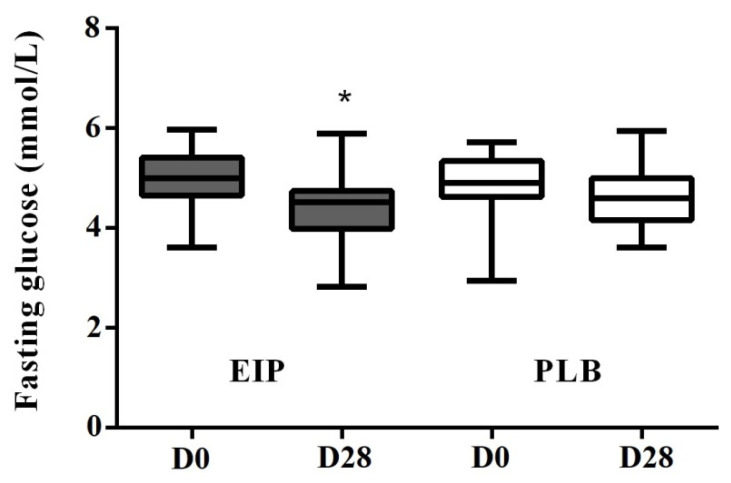
Effect of maté extract on fasting glycemia. Values are means ± SEMs (*n* = 34); D0 = value at the beginning of the period; D28 = value after 28 days of treatment. * Means between D0 and D28 in the EIP group were statistically different based on Tukey test comparisons (*p* < 0.05). (GraphPad Prism 6). EIP = *Ilex paraguariensis* extract; PLB = placebo.

As seen in Table 3, the HDL-c plasma concentration was not significantly different between the EIP and PLB treatments. However, when stratifying subjects according to Framingham risk score we noticed that the intermediate and high risk group (*n* = 15) presented an increase of HDL-c by +10%, (delta = +0.13 ± 0.05 mmol/L) when compared before and after the maté extract consumption (D0 = 1.30 ± 0.12 mmol/L versus D28 = 1.43 ± 0.12 mmol/L). In contrast, a decrease of HDL-c was observed in the placebo group (D0 = 1.43 ± 0.12 mmol/L versus D28 = 1.40 ± 0.12 mmol/L). The mix model showed no significant difference between the EIP and PLB treatments for fasting glucose (Table 3). However, attention was drawn to the mean values of fasting glucose before and after the treatment with EIP which were significantly different, with a variance test revealing a significant effect of EIP (*p* < 0.0002) regarding fasting glucose (delta = −0.57 ± 0.11 mmol/L). This difference between D0 and D28 was not observed after placebo consumption (Figure 3). 

**Figure 4:**
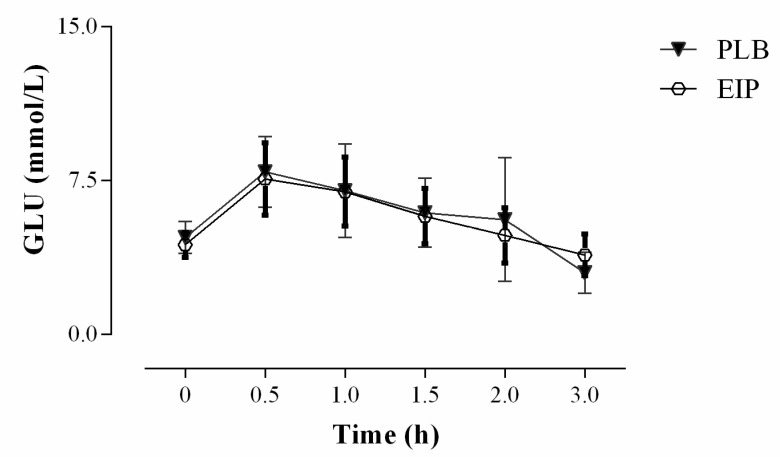
Kinetic curves of plasma glucose after oral glucose tolerance test (OGTT) carried out at the end of each experimental period. EIP = *Ilex paraguariensis* extract; PLB = placebo; (*n* = 34) (GraphPad Prism 6, Statistical Software).

To assess the impact of the intervention with maté on glucose tolerance in the study population, an oral glucose tolerance test (OGTT) was performed at the end of each treatment period. After the OGTT, the glucose concentrations were measured at T + 0.5, +1, +1.5, +2, and +3 h and the area under the curve (AUC) was calculated. As shown in Figure 4, after the glucose load we did not observed any statistically significant difference between treatment in the kinetic curves of glucose, as well as in the AUC between the EIP and PLB interventions (AUC (mmol/L × h): EIP = 19.57 ± 3.24; PLB = 20.61 ± 5.17; EIP-PLB = −1.14 ± 0.98; *p* value = 0.25).

### 3.3. Endothelial and Inflammatory Parameters

A subgroup of 12 volunteers, with FRS profiles representative of the entire study population (including 7 at low risk, 4 moderate risk and 1 high cardiovascular risk) was used to conduct a pilot study to provide preliminary data about the impact of the maté extract consumption on soluble biomarkers of inflammation (CRP, IL-6) and endothelial dysfunction (ICAM-1, VCAM-1).

The quantification of CRP, ICAM-1, VCAM-1 and IL-6 was done on plasma samples collected before (D0) and after the dietary intervention (D28) and also after the OGTT test (D28+) (Table 4). The CRP concentration was not significantly affected by the two treatments when measured in fasted conditions, while it decreased by half after the OGTT in both the PLB and EIP groups. The plasma levels of the cell adhesion molecules ICAM-1 and VCAM-1 did not change significantly in response to intervention under fasting condition as well as after the OGTT. IL-6 levels in fasting conditions were significantly reduced after the maté intervention (−19%), while no change was observed in the placebo group. By contrast the IL-6 response to the OGTT was similar between the PLB and EIP groups.

## 4. Discussion

As far as we know, this is the first randomized controlled trial that assess the impact of a well characterized maté extract providing a daily intake of phytochemicals in agreement with that consumed from traditional maté beverages on a range of cardiometabolic features. Like coffee, the major phytochemicals in maté are chlorogenic acids (CGAs) belonging to the caffeoylquinic acids (CQA) subclass [16]. However, maté clearly distinguishes from coffee by its richness in di-CQAs, a subgroup which has been yet little explored regarding both its bioavailability and bioefficacy in humans [16]. Based on a previous study which has evaluated the amount of caffeoylquinic acid compounds ingested daily from the consumption of traditional maté chimarrão beverage (512.5 to 1708.5 mg/day of total CGAs) [28], the capsules of dry maté extract used in the present trial provided daily 581 mg of total CGAs, with di-CQAs as the predominating forms. 

Hypoglycemic effects have been reported in animal and human studies for a range of dietary polyphenolic compounds administered as purified compounds or as polyphenol rich foods [4], including CGAs and coffee. The effects of CGAs have been related to an inhibition of carbohydrate digestion by inhibiting amylase [30,31], to the preventing action of glycosidase in the brush border of the small intestine [32], to modulation of gastrointestinal peptides shifting glucose absorption to more distal region in the GI tract [10] and also to a reduction of hepatic glucose output [33]. Recent investigations have suggested that CGA could regulate glucose metabolism by directly interacting with the AKT (protein kinase B) related pathway, leading to glycogen synthase activation and lowering blood glucose [34]. Based on this literature, we aimed to evaluate the impact of a nutritional intake of CQAs from maté extract on glycemic control in humans. Fasting blood glucose constitutes the main indicator to reflect the efficacy of glycemic control. The main finding from this trial carried out on a population of men at predisposed to cardiovascular risk is that consumption of the maté extract for four weeks caused a significant decrease in fasting blood glucose when values are compared before and after the dietary intervention (Figure 3). However, this hypoglycemic effect of maté extract was no longer observed under more stringent conditions, when comparing placebo versus extract treatments. This discrepancy could be explained by the low baseline glucose values of the study population at inclusion, making it difficult to detect significant differences between experimental placebo and maté groups. In any event, even if the magnitude of the reduction of fasting glucose we observed after mate extract consumption cannot have any clinical impact, it draws attention on the capacity of EIP to modulate glucose metabolism. This effect could reach a clinical relevance in individuals with abnormal fasting glucose values; however; this remains to be addressed specifically in a study population constituted by diabetics or prediabetic subjects. The shape of the plasma glucose curve after an OGTT is known to depend on insulin sensitivity and insulin secretion [35]. In the present study, the four-weeks intervention with maté extract did not induced changes in the response to OGTT compared to the placebo group. In both cases, the return to normality starting after 30 min and complete after 2 h reflected the good glucose homeostasis of the study population. In the present study, the reduction in fasting glucose in the maté group after four-week consumption is observed in a population mainly constituted by normoglycemic subjects, suggesting that this effect could be of particular relevance and of higher magnitude in a population of diabetic or prediabetic subjects. According to this hypothesis, a previous pilot study showed that Maté tea consumption (1 L/day providing 220 mg CGAs) during 60 days improved the glycemic control in T2DM subjects by reducing the fasting blood glucose by 17% [25]. 

A previous trial conducted in overweight adults reported that daily consumption of a CQAs rich coffee (provided 369 mono-CQAs mg/serving) induced a significant decrease in body weight and waist circumferences after 12 weeks [36]. This effect could be related to the increased postprandial energy expenditure and fat utilization observed in healthy humans after consuming daily 329 mg CGAs [37]. In our four-weeks study enrolling volunteers with a quite similar anthropometric profile and receiving daily 517 mg of total CQAs (including 238 mg as mono-CQAs), we did not observe any impact of the intervention on body weight or waist circumference. This could be due to the lower duration of our study, as supported by another trial which also lasted four-weeks and provided same results as ours [38]. In addition, a human trial using supplements of yerba-maté (3 g/days but without any specifications regarding the CQAs dosage) reported anti-obesity effects after 12 weeks of supplementation [23]. 

Several studies performed in hypertension animal models or in hypertensive subjects have reported a hypotensive effect in response to CGAs consumption which could result from the capacity of CGAs to regulate vasomotor agents and vasoconstrictor enzymes and to reduce oxidative stress [8]. In the present trial, as well as in a previous one studying the hypotensive effects of CQAs [39], which did not select a study population with blood pressure classified as greater or equal to stage I hypertension, no blood pressure lowering effect of the intervention could have been detected.

The in vivo effect of CGAs on dyslipidemia has only been evaluated in animals. Available supplementation studies indicated improvements of the serum lipid profiles in rodents fed a high fat or high cholesterol diet [40,41,42], with a hypocholesterolemic effect suggested to be mediated through the up-regulation of PPAR-α expression by CGAs [42]. An improvement in blood cholesterol profile with maté was previously reported in dyslipidemic individuals on statin therapy consuming yerba-maté infusion (990 mL/d) with a significant decrease of LDL-c (−8.6%) and increase of HDL-c (+6.2%) after 40 days [26]. Another pilot study conducted on diabetics and pre-diabetic, consuming during 60 days the same daily dose of yerba-maté infusion as previously, also showed a significant decrease in the levels of (LDL-c) [25]. In our study, in which population well-balanced between normo and dyslipidemic subjects, the four-weeks period of maté extract intake did not induce any significant improvement in mean values of plasma cholesterol and TAG. However, after stratification of the subjects according to their Framingham risk score, the intermediate and high risk group (*n* = 15) showed a 10% increase of HDL-c from baseline after maté extract consumption, whereas no difference was observed after placebo. This result suggests that consumption of CGAs through maté could be of particular interest to regulate lipemia in dyslipidemic subjects. However, this deserves further investigations focusing on a study population presenting the appropriate characteristics. 

A glucose load was previously reported to induce transient endothelial dysfunction in healthy adults [43]. Based on this study and in order to provide the first insight into the potential for sub-chronic consumption of maté in attenuating a transient endothelial dysfunction, we evaluated some markers related to endothelial dysfunction (cell adhesion molecules: VCAM-1, ICAM-1) and inflammation (IL-6 and CRP) in the blood sampled from a representative subset of our study population in fasted conditions and after the OGGT (Table 4). In contrast to which was initially expected, no change in adhesion molecules levels have been detected in response to the glucose load. This result suggesting the absence of transient endothelial dysfunction is in agreement with Major-Pedersen et al., 2008 [44] who reported that in healthy individuals with normal values of insulin and glucose the oral glucose load does not induce endothelial dysfunction in postprandial conditions. Interestingly, our results indicated that a four-weeks intake of maté extract significantly reduced (by −20%) fasted plasma levels of IL-6, a cytokine tightly involved in the metabolic regulation of CRP. As recently reviewed [45], data from animal and cell studies indicated that mono-CQAs can mitigate inflammation through inhibition of pro-inflammatory cytokines via modulation of key transcription factors; however, no data are available regarding specifically di-CQAs. This literature together with our preliminary result suggest that maté CGAs could participate in the modulation of chronic low grade inflammation in humans, however these aspects should be addressed in further specific studies.

To conclude, this study focused on the impact of CGAs from maté at a daily dose achievable through traditional maté beverages intakes on some metabolic and inflammatory parameters known to be critical for a population of middle age and overweight individuals at low to moderate risk of CVD. It has provided the first clinical insights about the interest of maté for this population group, however, in most case we observed trends that have not yet been confirmed. This limitation could have several origins. First, the duration of the study was probably too short to be able to detect changes in some parameters that could have been observed after a longer intervention time. The study population was selected based on its predisposition to cardiovascular risk. However, the mean baseline values were in the normal range for most of the parameters assessed, making it difficult to observe significant improvement in response to the intake of a nutritional dose of maté CQAs. In agreement with that, the observed trends or significance have been reached only after stratification according to the Framingham risk score, suggesting a higher benefit from maté consumption in subject at risk of CVD. However, this hypothesis needs to be addressed in specific trials.

## Figures and Tables

**Table 1:** Quantification of phytochemicals in the *Ilex paraguariensis* extract used in the study.

Compounds	% in Dry Extract (*w*/*w*)	Amount per Capsule (mg) *	Daily Intake (mg)
3-O-caffeoyl quinic acid	4.91	12.28	110.49
5-O-caffeoyl quinic acid	3.19	7.98	71.82
4-O-caffeoylquinic acid	3.87	9.67	87.07
∑-Mono-O-CQAs	11.97	29.93	269.38
3,4-Dicaffeoylquinic acid	2.97	7.44	66.93
3,5-Dicaffeoylquinic acid	4.40	10.99	98.95
4,5-Dicaffeoylquinic acid	6.49	16.23	146.14
∑-Di-O-CQAs	13.87	34.67	312.01
∑-Mono and Di-O-CQAs	25.84	64.60	581.39
Caffeine	3.32	8.32	74.87
Theobromine	0.35	0.88	7.93
∑ Methylxanthines	3.67	9.2	82.8

(*) caps = 250 mg extract; CQAs: caffeoyl quinic acids.

**Table 2:** Characteristics of the study population on the day of screening (*n* = 34).

Characteristics	Mean ± SD	Range	Normal Values
Age, y	50.11 ± 4.59	45–63	-
BW, kg	82.08 ± 12.13	57.6–106.4	-
WC, cm	93.94 ± 9.40	75–113	<94 ^(b)^
BMI, kg/m^2^	27.28 ± 3.28	20.0–33.5	20 to 25 ^(b)^
PUL, b.p.m	69.20 ±15.63	40–136	60–100 ^(c)^
SBP, mm Hg	122 ± 12.40	98–160	120 ^(b)^
DBP, mm Hg	80 ± 11.81	60–116	80 ^(b)^
Triglycerides, mmol/L	1.61 ± 0.93	0.66–4.55	1.7 ^(b)^
Total cholesterol, mmol/L	5.57 ± 0.58	3.72–8.04	<4.0 ^(a)^
HDL cholesterol, mmol/L	1.38 ± 0.34	0.96–2.28	≥1.5 ^(a)^
LDL cholesterol, mmol/L	3.39 ± 0.89	1.75–5.51	<2.6 ^(b)^
GLU, mmol/L	5.22 ± 0.55	4.06–6.42	4.9–5.3 ^(d)^
Creatinine, µmol/L	84.76 ± 19.08	-	-
Urea, mg/dL	34.03 ± 7.79	-	-
Alanine Transaminase, U/L	24.20 ± 7.83	-	-
Aspartate. Transaminase, U/L	21.97 ± 7.66	-	-
Framingham risk score, %	11.04 ± 5.73	3.30–25.30	<10% ^(e)^

^(a)^ American Heart Association, About Cholesterol. https://www.heart.org/en/health-topics/cholesterol/about-cholesterol. Accessed on 24 May 2020. ^(b)^ European Society of Cardiology, ESC/EAS Guidelines for the management of dyslipidaemias: lipid modification to reduce cardiovascular risk. https://www.escardio.org/Guidelines. Accessed on 24 May 2020. ^(c)^ American Heart Association, All About Heart Rate (Pulse). https://www.heart.org/en/health-topics/high-blood-pressure/the-facts-about-high-blood-pressure/all-about-heart-rate-pulse. Accessed on 24 May 2020. ^(d)^ World Health Organization, Global report on diabetes. World Health Organization, https://apps.who.int/iris/handle/10665/204871. Accessed on 24 May 2020. ^(e)^ Canadian Cardiovascular Society, Canadian guidelines for the diagnosis and treatment of dyslipidemia and prevention of cardiovascular disease in the adult—2009 recommendations. https://www.ccs.ca/en/guidelines/guidelines-library. Accessed on 24 May 2020.

**Table 3:** Effects of the intervention on hemodynamic, anthropometric, and biochemical parameters ^1^.

	Baseline	EIP	PLB	EIP-PLB	*p* Value ^2^
SBP, mm Hg	121.56 ± 10.97	119.57 ± 10.21	121.08 ± 8.92	−1.516	0.224
DBP, mm Hg	76.78 ± 12.32	76.06 ± 12.12	77.52 ± 12.12	−1.463	0.195
PUL, bpm	65.38 ± 7.85	66.74 ± 8.08	67.32 ± 9.19	−0.588	0.693
WC, cm	93.46 ± 9.14	93.73 ± 9.40	93.51 ± 8.48	0.214	0.702
BW, kg	81.99 ± 12.24	81.76 ± 12.08	81.96 ± 11.93	−0.196	0.364
BMI, kg/m^2^	27.34 ± 3.51	27.17 ± 3.37	27.24 ± 3.43	−0.065	0.360
TC, mmol/L	5.16 ± 0.90	4.97 ± 1.25	4.94 ± 1.15	0.032	0.881
HDL-c, mmol/L	1.35 ± 0.33	1.41 ± 0.40	1.34 ± 0.31	0.066	0.20
LDL-c, mmol/L	3.02 ± 0.81	2.95 ± 1.07	2.89 ± 0.93	0.0567	0.748
TGY, mmol/L	1.70 ±1.01	1.51 ± 0.86	1.63 ± 0.98	−0.117	0.348
GLU, mmol/L	4.95 ± 0.44	4.42 ± 0.63	4.60 ± 0.52	−0.172	0.132

^1^ All values are means ± SD (*n* = 34). Mixed model are adjusted for baseline value. EIP, *Ilex paraguariensis* extract; PLB, placebo. ^2^ P value for treatment effect (PROC MIXED, SAS Institute).

**Table 4:** Plasma levels of cytokines and adhesion molecules in response to the intervention and after glucose challenge.

	EIP			PLB		
	D0	D28	D28+	D0	D28	D28+
CRP (mg/dL)	1.13 ± 0.35 ^a^	1.070 ± 0.25 ^a^	0.50 ± 0.18 ^b^	1.15 ± 0.34 ^a^	0.82 ± 0.26 ^a^	0.60 ± 0.25 ^b^
ICAM-1 (ng/mL)	532.6 ± 46.54	523.60 ± 55.07	474.9 ± 58.12	444.2 ± 56.68	469.20 ± 72.94	456.00 ± 63.97
VCAM-1 (ng/mL)	478.5 ± 43.09	535.3 ± 67.16	467.19 ± 50.36	478.9 ± 63.87	459.6 ± 60.64	480.4 ± 69.85
IL-6 (pg/mL)	1.71 ± 0.26 ^a^	1.39 ± 0.17 ^b^	1.73 ± 0.16 ^ab^	1.51 ± 0.22	1.20 ± 0.22	1.78 ± 0.45

Values are means ± SD, (*n* = 12). EIP = *Ilex paraguariensis* extract; PLB = placebo. D0 = values at the beginning of period, fasting; D28 = values at 28 days after consumption of capsules, fasting; D28+ = values at 28 days after capsules consumption and T + 1.0 h (CRP), T + 2.0 h (ICAM-1, VCAM-1) and T + 3.0 h (IL-6) after the oral glucose load. Tukey test comparisons (*p* < 0.05), line notations represent significant differences.
